# An Experimental Study on Structural Concrete Containing Recycled Aggregates and Powder from Construction and Demolition Waste

**DOI:** 10.3390/ma15072458

**Published:** 2022-03-26

**Authors:** Jeonghyun Kim, Anna M. Grabiec, Andrzej Ubysz

**Affiliations:** 1 Faculty of Civil Engineering, Wrocław University of Science and Technology, 50-370 Wrocław, Poland; andrzej.ubysz@pwr.edu.pl; 2 Faculty of Environmental Engineering and Mechanical Engineering, Poznań University of Life Sciences, 60-649 Poznań, Poland; agra@up.poznan.pl

**Keywords:** recycled coarse aggregate, recycled fine aggregate, recycled powder, recycled aggregate concrete, construction and demolition waste

## Abstract

For complete utilization of construction and demolition (C&D) waste, an investigation of all size fractions of C&D waste generated during the recycling process should be conducted. In this work, the effects of three recycled concrete materials with different sizes (recycled coarse aggregate (RCA) with a size of 4.75–25 mm, recycled fine aggregate (RFA) of 0.15–4.75 mm, and recycled powder (RP) smaller than 0.15 mm) produced from concrete waste on the fresh and hardened mechanical properties of concrete were evaluated. The replacement ratios of natural coarse and fine aggregates by RCA and RFA were 30, 60, and 100%, and those of ordinary Portland cement for RP were 10, 20, and 30%. The results showed that the concrete properties deteriorated with increasing replacement ratio regardless of the type of recycled materials. The properties were reduced in the order of the use of RFA, RCA, and the simultaneous use of RCA and RFA. In addition, concrete with 30% RP showed lower mechanical strength than concrete with 100% RCA and 100% RFA. However, all concretes could be applicable for structural purposes under different environmental exposure conditions. In particular, concretes with 10% RP and 20% RP showed better cost-benefits compared to natural aggregate concrete with 100% ordinary Portland cement. These promising findings provide valuable initiatives for the effective and complete recycling of C&D waste.

## 1. Introduction

The evidence for the loss of stability of the Earth’s natural climate system, especially in view of the increase in the frequency of extreme weather phenomena including global warming, despite the debate over their actual causes (solar activity, Milankovitch cycles, volcanic activity, and El Niṅo Southern Oscillation phenomenon) is difficult to contest. However, the most probable main cause is the growing increase of carbon dioxide (CO_2_) in the atmosphere, mainly due to anthropogenic activities. One of the areas of human activity involved in the increase of CO_2_ emission is the concrete industry. Despite the efforts of several researchers, such as carbon-friendly design and extending the lifecycle of structures to reduce CO_2_ emissions [1,2], there is no doubt that concrete will remain a basic construction material for a long time. Unfortunately, its production focused on high quality imposes a heavy burden on the environment. A major contributor to the environmental impact for energy consumption and emission of greenhouse gases, including CO_2_, is the production of cement, the key constituent of concrete. According to Andrew [3], the global CO_2_ emissions accompanying the cement production in 2016 were estimated at 1.45 ± 0.20 Gt CO_2_. However, other technological operations related to the production of concrete, from the extraction of natural resources (natural aggregates, raw materials for processing into crushed aggregates, raw materials necessary to produce cement), their transport, through mixing the components of the concrete mixture, its laying and compaction, including concrete care as well as the use and maintenance of buildings until the last stages of their life, also make a significant contribution to the anthropogenic CO_2_ emissions [4].

It is also important to note that the deposits of natural resources are depleting. Their place in natural space is taken by waste materials from various production, the effect of which is a violation of ecological systems [3]. The main source of waste generated in the world is construction activities. Construction and demolition (C&D) waste in 40 countries reached more than 3 billion tons per year [5], and China and the United States are reported to be responsible for about 30% of the global C&D waste generation [6]. Furthermore, many studies have shown that C&D waste accounts for 30–50% of the total waste generated worldwide, and the amount of C&D waste is gradually increasing annually [7,8,9]. Therefore, it is rational to reutilize C&D waste, particularly from concrete, such as recycled coarse aggregate (RCA), recycled fine aggregate (RFA), and recycled powder (RP), even using repeated crushed recycled aggregate [10,11,12]. The performance of concrete with recycled aggregates is obviously worse. However, when asked what is more important, the performance of the concrete which can be improved in various ways, or environmental protection for ecological reasons, understood in a wide range, the second approach is more justified.

In this context, the recyclability of C&D waste has been investigated. One of the applications considered as advanced recycling is the replacement of natural materials with recycled materials in concrete production. In general, three recycled concrete materials can be obtained from C&D waste, depending on the particle size, i.e., RCA, RFA, and RP (hereinafter the recycled concrete materials in this paper refer to RCA, RFA, and RP).

RCA, which occupies the largest volume in concrete, has received the most attention from researchers as a substitute for natural coarse aggregates (NCA). In addition, technologies and methods for improving the performance of recycled aggregate concrete (RAC) have been developed mainly based on RCA. Therefore, the knowledge system on the effects of RCA on concrete is well-established [13,14], and guidelines for the use of RCA have been suggested by several countries [8,15,16]. Some researchers pointed out that research on concrete recycling has been relatively limited to RCA [17,18]. RFA as a substitute for river sand and natural fine aggregate (NFA) has received relatively less attention compared to RCA. Recent environmental issues, such as restrictions on sand mining in some areas for ecosystem protection, urge the use of RFA. In the past, the use of RFA in concrete was restricted due to concerns about material contamination and difficulties in quality control [19], but recent studies have reported that RFA does not seriously affect the mechanical and durability properties of concrete within an appropriate replacement ratio [20,21,22]. Replacing both NCA and NFA with RCA and RFA can yield higher energy and resource savings because a greater amount of recycled concrete materials produced can be used, but the simultaneous use of RCA and RFA has a more negative impact than the use of a single type of the recycled concrete material. In a study performed by Pedro et al. [23], the 28-day compressive strength of concrete made with 100% RCA and concrete made with 100% RFA was 5.4% and 9.9% lower than that of control concrete, respectively, whereas that of concrete with 100% RCA and 100% RFA decreased by 14.9%. Although some previous studies have been published [23,24], the simultaneous incorporation of RCA and RFA into concrete is still a new area in which limited scientific research has been conducted. Therefore, the obtained results have important implications both in terms of scientific and practical use. Lifecycle assessment showed that cement production was the largest contributor in all environmental impact categories, irrespective of natural aggregate concrete (NAC) and RAC [25,26]. Hence, reducing cement consumption is a clear alternative to reducing CO_2_ emissions in the concrete industry. Accordingly, several studies have investigated the effectiveness of various supplementary cementitious materials, such as glass powder and fly ash, to decrease cement usage [27,28]. However, RP as a supplementary cementitious material is arguably the least investigated material compared to RCA and RFA [18]. In the concrete matrix, RP acts as a filler that fills the pores of the concrete and makes it compact, but on the other hand, the low reactivity of RP does not contribute to strength development by forming fewer hydration products, which is the cause of the low performance compared to concrete made of Portland cement.

In this context, the transition to the ‘zero-waste’ pursued by today’s society in the construction sector cannot be achieved without a systematic discussion of the influence of recycled concrete materials of all size fractions generated from C&D waste. As described above, several studies have been conducted on the application of RCA, RFA, and RP as concrete materials, but these studies discuss the properties of concrete using materials obtained from different C&D waste sources or using two recycled concrete materials, mainly RCA and RP [29,30,31]. Particularly, little research has been carried out on the properties of concrete incorporating RCA, RFA, and RP obtained from a single source of concrete waste. Thus, this study aims to fill the gap in scientific and technical understanding of the behavior of concretes which incorporate recycled concrete materials with various size fractions obtainable by C&D waste recycling. To achieve this objective, the effect on fresh and hardened mechanical properties of concretes made from each recycled material at various replacement ratios was investigated. The fresh-state properties studied included slump and air content, and the hardened properties were evaluated for compressive strength, tensile strength, and elastic modulus. Subsequently, the correlations between the hardened properties were compared with prediction models presented in the literature. In the end, the economic and environmental benefits of each mix were analyzed. This study can provide valuable insights on the economical and eco-friendly use of recycled concrete materials obtained from C&D waste for structural concrete.

## 2. Materials and Methods

### 2.1. Materials

The RCA, RFA, and RP used in this study were obtained by crushing intentionally produced NAC. This NAC serves both as a parent concrete for obtaining recycled concrete materials and as a reference concrete (RC) for property comparison. Natural granitic crushed aggregate with a nominal maximum aggregate size of 25 mm and siliceous river sand were used as NCA and NFA for the production of parent concrete. The specific gravity and water absorption of NCA were 2.68 and 0.88%, respectively, and those of NFA were 2.6 and 0.91%. Ordinary Portland cement (OPC) with a specific gravity of 3.14 and a specific surface area of 3550 cm^2^/g was used as a cementitious binder. The composition of RC is provided in Table 1. The target strength and target slump of RC were 30 MPa and 100 mm, respectively.

**Figure 1:**
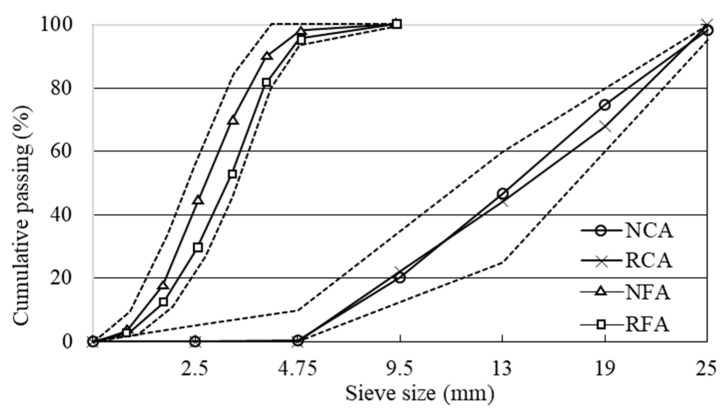
Particle size distribution of natural and recycled aggregates.

At 56 days of age, the RC specimens after the testing described in Section 2.3 were compressed with a hydraulic universal testing machine and crushed into large pieces (approximately 50 to 100 mm in length), and these concrete fragments were crushed into sizes (up to 25 mm) suitable for concrete production using the Los Angeles ball mill. By combining mechanical and manual sieving, three recycled concrete materials with different size fractions were obtained: RCA with a size of 4.75–25 mm, RFA with a size of 0.15–4.75 mm, and RP smaller than 0.15 mm [32]. Table 2 shows the properties of recycled aggregates along with natural ones. RCA and RFA are less dense than NCA and NFA due to their greater porosity, resulting in higher water absorption. Figure 1 plots the particle size distribution curves for each aggregate.

### 2.2. Mix Design and Testing Methods

To investigate the influence of RCA, RFA, and RP on the mechanical performance of concrete, 12 different concrete mixtures were prepared based on the ACI mix design [33]. According to [15], several regulations suggest that recycled aggregates (mainly recycled coarse aggregate) can replace about 20% up to 100% of natural aggregates. In addition, RCA can replace up to 60% of NCA when RCA is the only recycled material in concrete. When using RCA and RFA simultaneously, up to 30% of the total aggregate can be replaced [8].

Therefore, in this study, the replacement ratios of 30%, 60%, and 100% were applied. The description and notation for each mixture are as follows:

- RCAC-replacement ratio: concrete made from NCA, NFA, OPC, and RCA that replaces NCA in a certain ratio (i.e., 30%, 60%, and 100%).
- RFAC-replacement ratio: concrete made from NCA, NFA, OPC, and RFA that replaces NFA in a certain ratio (30%, 60%, and 100%).
- RPC-replacement ratio: Concrete made with NCA, NFA, OPC, and RP that replaces OPC in a certain proportion (10%, 20%, and 30%). Test results for RPC were adopted from the previous study of the author [32].
- RCFAC-replacement ratio: Concrete made by replacing both NCA and NFA with RCA and RFA in a certain percentage (30%, 60%, and 100%). OPC was used as a binder.

All concrete mixes had a constant quantity of 389 kg/m^3^ binder (i.e., sum of OPC and RP), and the water-to-cement (w/c) ratio was fixed at 0.45. Details of the mix proportions of concrete are shown in Table 3.

Since the moisture state of aggregates is one of the parameters influencing the properties of concrete, the moisture condition of each aggregate was considered before mixing. Due to the high water absorption of RCA, pre-wetting is required to obtain proper workability. However, RCA in the saturated surface dry (SSD) state did not produce a favorable effect in terms of mechanical properties of concrete [34,35]. On the other hand, for RFA, the results of previous studies have reported that fine aggregate in the SSD state was more favorable to the mechanical strength of concrete than in the air-dry and oven-dry state [36,37]. Therefore, NCA, NFA, and RFA were used in the SSD state, and partially saturated RCA dried at room temperature 24 h before mixing after complete saturation was used [38].

A mechanical pan mixer with a capacity of 60 L was used for the mixing of concrete components. Coarse and fine aggregates were put in a mixer and mixed for 30 s, then OPC was added and mixed for 90 s to disperse the material. Water was then added and mixed for 2 min. After the mixing process, the fresh properties of the concrete were evaluated, and specimens for evaluating the mechanical properties of the concrete were made in 100 × 200 cylindrical molds as per ASTM C192 [39]. The specimens were demolded 24 h after casting and cured in a container with tap water of 20 °C right before testing. Mechanical properties were measured using a hydraulic universal testing machine. Compressive strength and elastic modulus were measured at 28 and 56 days, and splitting tensile strength was measured at 28 days. Table 4 summarizes test types, standards, specimen sizes, and test ages.

**Table 4:** Summary of testing protocol.

Test	Standard	Specimen Size (mm)	Test Age (Days)
Fresh state			
Air content	[40]	n/a	Immediately after mixing
Consistency	[41]	n/a	Immediately after mixing
Hardened state			
Compressive strength	[42]	Ø100 × 200	28 and 56
Splitting tensile strength	[43]	Ø100 × 200	28
Elastic modulus	[44]	Ø100 × 200	28 and 56

### 2.3. Cost and Environmental Impact Assessment

A cost and an environmental impact assessment analysis were performed on the investigated concretes. The manufacturing cost per cubic meter (UDS/m^3^) of each concrete was calculated based on the raw material price surveyed by the Construction Association of Korea as of November 2021 (Table 5). Based on the 28-day compressive strength test results, the strength–cost value analysis of each mix was discussed.

Based on the mix proportions, the global warming potential (GWP) of each mix was assessed. In Table 5, CO_2_ equivalent emissions per kilogram (kg CO_2_-eq./kg) from the manufacture of concrete components are presented and the values have been taken from [24,45,46].

**Table 5:** Unit price and global warming potential of concrete components.

Materials	Unit Cost (USD/ton)	GWP (kg CO_2_-eq./kg)
OPC	103.75	0.931 [46]
RP	4.15	0.2457 [46]
NCA	8.67	0.0244 [45]
NFA	9.58	0.025 [45]
RCA	4.15	0.00744 [45]
RFA	6.64	0.012 [24]

## 3. Results and Discussion

### 3.1. Fresh Properties

**Figure 2:**
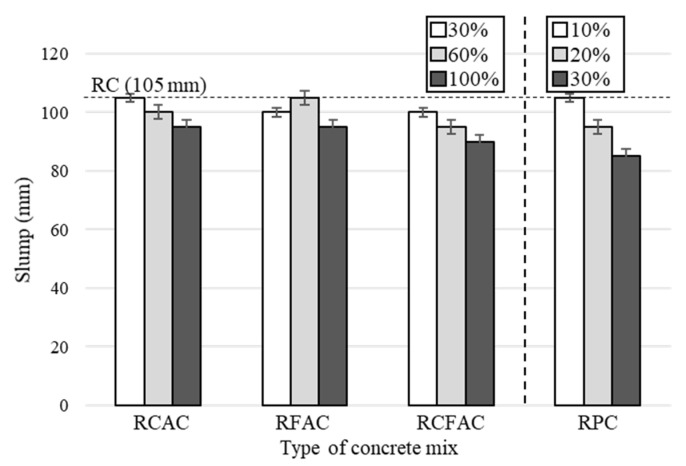
Slump test of concrete mixes made of recycled aggregates and recycled powder.

Figure 2 shows the results of the slump test for RCAC, RFAC, and RCFAC at replacement ratios of 30%, 60%, and 100%, and those for RPC at replacement ratios of 10%, 20%, and 30%. Some mixes (RCAC-30, RFAC-60, RPC-10) showed the same slump value of 105 mm as RC, but overall, the slump was on a downward trend as the replacement ratio of each recycled concrete material increased. This result was generally observed in previous studies that investigated concrete containing recycled aggregates and RP, and is due to the high water absorption of recycled concrete materials compared to natural materials [47]. For each type of concrete mix, the maximum slump loss was observed at a replacement ratio of 100% (30% for RPC). In comparison with RC, the slump loss of RCAC and RFAC was up to 10%, and that of RCFAC was 14%. The slump of the concrete mix containing RP decreased by 10% and 19% at the replacement ratios of 20% and 30%, respectively, showing greater slump loss than RCAC, RFAC, and RCFAC. This may be because, unlike aggregates to which pre-wetting was applied, the moisture state of RP was not considered. Nevertheless, all mixes were within the tolerance of ±25 mm for concrete with a target slump of 100 mm according to ASTM C94 [48] (i.e., from 75 to 125 mm). Therefore, in order to obtain workability similar to that of RC, moisture compensation such as pre-wetting and the addition of mixing water should be considered.

**Figure 3:**
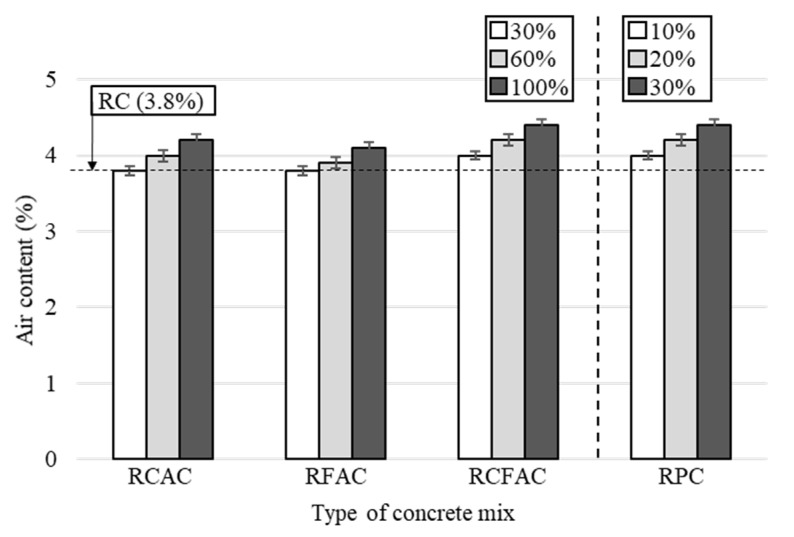
Air content of concrete mixes made of recycled aggregates and recycled powder.

The test results for the air content of the concrete mixes are shown in Figure 3. The measured air content was within the tolerance of 4.5% ± 1.5% according to the ASTM C94 [48]. The air content of RC was 3.8%, and the concretes used with recycled concrete materials exhibited higher air content than the RC due to the porosity of the recycled materials. RFAC showed a relatively low increase in air content compared to RCAC and RPC. This is because the pores of RFA used in SSD conditions were filled with water, contributing to suppression of the increase in air content [49].

### 3.2. Hardened Properties

#### 3.2.1. Compressive Strength

**Figure 4:**
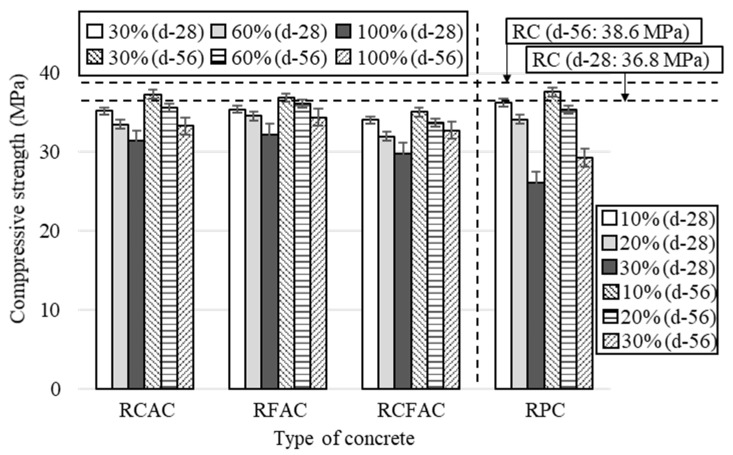
Test results of compressive strength of concretes made of recycled aggregates and recycled powder.

The 28- and 56-day compressive strengths for different types of concrete incorporating each recycled concrete material are shown in Figure 4. The replacement ratios for RCAC, RFAC, and RCFAC were 30, 60, and 100%, while those of RP were 10, 20, and 30%.

The experimental results have shown that the addition of recycled concrete materials reduces compressive strength regardless of the type of recycled material. This is in line with the general consensus of previous studies that the use of recycled concrete materials has a negative effect on the mechanical properties of concrete [50,51,52,53]. In comparison to RC, concretes with 30%, 60%, and 100% RCA showed 4%, 9%, and 15% losses in compressive strength at 28 days, respectively, and the losses at 56 days were 3%, 8%, and 14%. The reduction in compressive strength by replacing NFA with RFA was slightly lower than when replacing NCA with RCA. The 28-day compressive strength of RFAC was 4%, 6%, and 12% lower than that of RC at 30%, 60%, and 100% replacement ratios, but 0.2, 1.1, and 0.8 MPa higher than that of RCAC. This may be because the volume occupied by fine aggregates in unit concrete is smaller than that of coarse aggregates. However, there are conflicting results as to which of RCA or RFA has a more dominant effect on the poor performance of concrete. A greater loss of strength was observed in concrete with RCA than in concrete with RFA in some studies [54,55,56], and vice versa in other studies [17,23].

For concrete incorporating RP, the difference in compressive strength between RPC-10 and RC was 0.5 and 1 MPa at 28 days and 56 days, which is only 1% and 3% lower than that of RC. This insignificant variation was attributed to the filling effect, whereby RP, which is finer than NFA, fills the micropores, where the concrete becomes more compact and dense, reducing internal stresses and early crack propagation [57]. However, as the RP content increases, the negative effects of reduced hydration products outweigh the positive effects of filling [58]. The compressive strength of concrete with 20% RP as a cement binder was lower than that of RCAC-60, RFAC-60, and RCFAC-30. In addition, the maximum loss of compressive strength for all concretes investigated was observed in RPC-30, and the losses were 29% and 24% at 28 days and 56 days, respectively. This is in the range of losses reported in the studies of Xiao et al. [51] (7.7% reduction in strength at 30% RP replacement ratio) and Cantero et al. [59] (40% decrease in strength at 25% replacement ratio).

**Figure 5:**
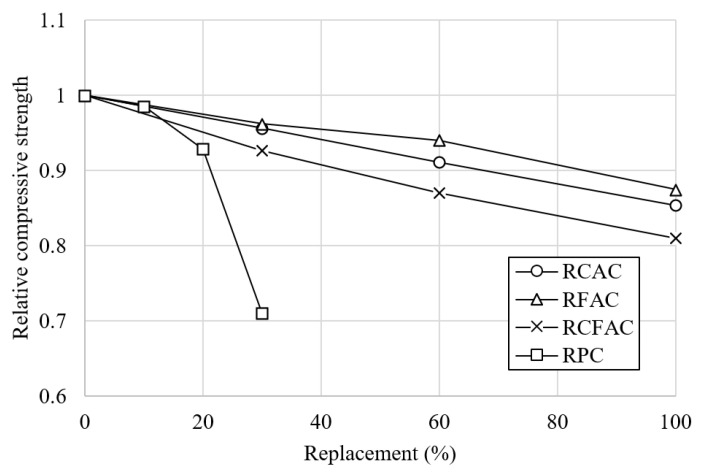
Relative compressive strength of various concretes by replacement ratio.

Figure 5 shows the behavior of the relative compressive strength of concretes made from different recycled concrete materials as a function of the replacement ratio. The strength decreased in the order of RCAC, RFAC, and RCFAC at the same replacement ratio. Concrete with simultaneous incorporation of RCA and RFA has a greater loss of compressive strength than concrete with RCA or RFA, which can be clearly seen in Figure 5. At a 100% replacement ratio, the compressive strength of RCAC, RFAC, and RCFAC decreased by 12–19%. This value is similar to or slightly lower than the loss reported in previous studies [21,23,54,56]. Khatib [21] reported a 36% reduction in compressive strength in concrete made with 100% RFA, and Guo et al. [54] reported a strength loss of up to 42.2% in concrete using both 100% RCA and 100% RFA. On the other hand, Cabral et al. [56] reported that the simultaneous use of 100% RCA and 100% RFA reduced the compressive strength by only 6–19%, and Pedro et al. [23] also reported a similar decrease in strength of 8–16%.

Table 6 briefly summarizes the minimum required compressive strength of concrete by environmental exposure classes specified in PN-EN 206:2016 [60]. With the increase in compressive strength, concrete can be applied in moderate to aggressive environments. Concrete with a compressive strength of less than 8 MPa cannot be used in environments where there is a risk of corrosion, whereas concrete with a compressive strength of over 35 MPa can be used as structural concrete in very harsh environments with constant or frequent exposure to seawater, carbonation, and sulfates. Among the concretes investigated in this study, RC, RCAC-30, RFAC-30, and RPC-10 mixtures can be used in all environments, and RCAC-60, RCAC-100, RFAC-60, RFAC-100, RCFAC-30, RCFAC-60, and RPC-20 are feasible options as structural concrete under no seawater exposure. RPC-30, which has the lowest compressive strength of 26.1 MPa, can also be used as structural concrete under moderate freezing-and-thawing attack.

#### 3.2.2. Splitting Tensile Strength

**Figure 6:**
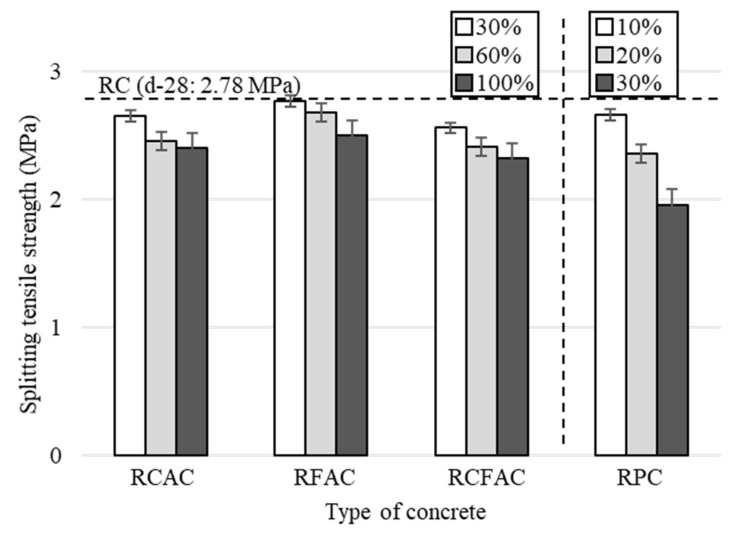
Test results of splitting tensile strength of concretes made of recycled aggregates and recycled powder.

The test results for splitting tensile strength at 28 days are presented in Figure 6. The results showed a similar trend to the compressive strength test result. That is, regardless of the concrete type, splitting tensile strength decreased as the content of recycled concrete materials increased. The 28-day splitting tensile strength of RC was 2.78 MPa, and those of recycled concretes varied (between 2.4 and 2.65 MPa for RCAC, 2.5–2.77 MPa for RFAC, 2.32–2.56 MPa for RCFAC, and 1.96–2.66 MPa for RPC). The loss of tensile strength in concrete made from RCA and RFA, which is low-strength and porous compared to NCA and NFA, is caused by poor bonding in the interfacial transition zone between the aggregate and cement paste [61]. On the other hand, for RPC, a decrease in strength with RP content occurs because RP, which has lower reactivity than cement, does not contribute to the strength development of concrete [62]. 

**Figure 7:**
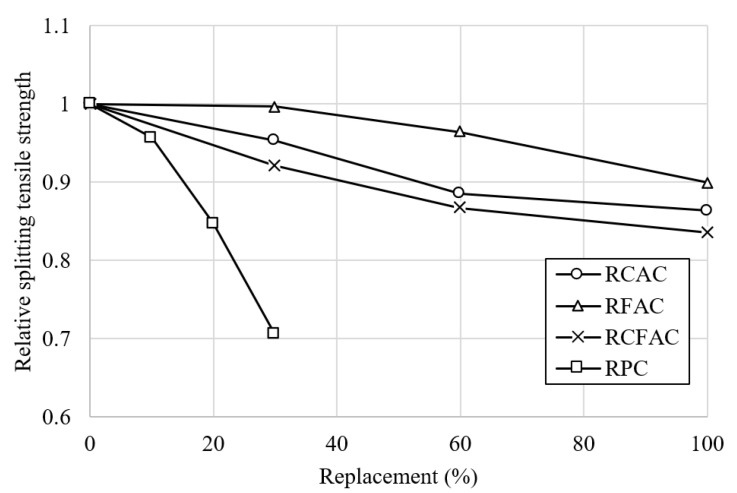
Relative splitting tensile strength of various concretes by replacement ratio.

Figure 7 shows the relative splitting tensile strength of concretes as a function of the replacement ratio. At the same replacement level, the strength decreased in the order of RFAC, RCAC, and RCFAC, with reductions of 5–14%, 0–10%, and 8–16%, respectively, in comparison with RC. Therefore, RCA seems to have a more negative effect on the tensile strength of concrete than RFA. The maximum reduction in tensile strength was observed for RCFAC-100, the concrete incorporating 100% RCA and 100% RFA simultaneously, which is consistent with the results of a previous study conducted by Singh et al. [17]. 

The addition of 10% RP did not seem to have a significant effect on the splitting tensile strength of concrete. However, when 20% RP was added, tensile strength dropped sharply and showed similar strength to that of RCFAC-100. The tensile strength loss of RPC observed in this study was 4–29%, which is in good agreement with the values reported in previous literature. Cantero et al. [59] reported a decrease in tensile strength of 19.9% at 25% RP replacement, and Xiao et al. [51] and Kim [62] reported reductions of 10.6% and 21%, respectively, at the 30% replacement rate.

#### 3.2.3. Elastic Modulus

**Figure 8:**
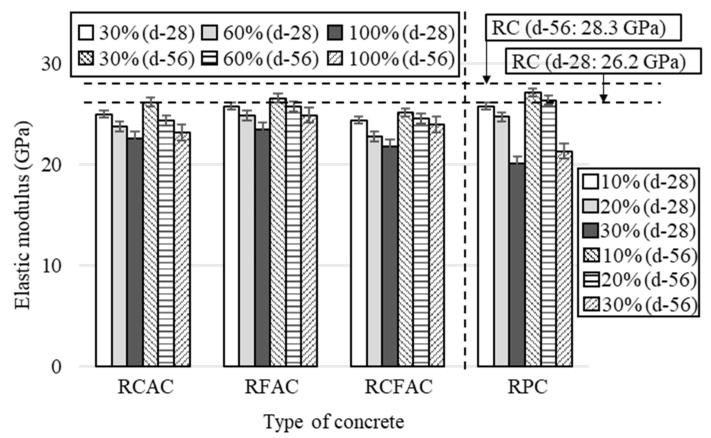
Test results of elastic modulus of concretes made of recycled aggregates and recycled powder.

**Figure 9:**
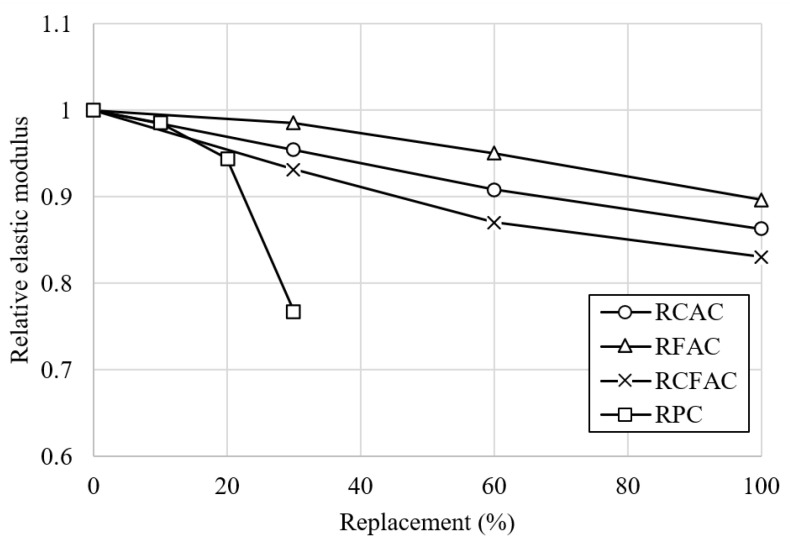
Relative elastic modulus of various concretes by replacement ratio.

The test results of the elastic modulus of concretes at 28 and 56 days are presented in Figure 8, and Figure 9 shows the relative elastic modulus at 28 days.

Since the elastic modulus of concrete is closely related to the stiffness of the aggregate, porosity, and bonding of mortar, Evangelista and de Brito [63] noted that low levels of RA replacement do not cause a significant loss of elastic modulus. This supports that RFAC has the lowest modulus loss because it has the smallest volume of recycled material replacing natural materials in unit concrete. Compared to RC, the elastic modulus of RFAC decreased by 2–10%, while RCAC and RCFAC decreased by 5–14% and 7–17%, respectively. In a study by Cabral et al. [56], the elastic modulus of concrete made with 100% RCA decreased by 21%, while that of concrete made with 100% RFA decreased by 10%. For the type of concrete in which natural aggregates were replaced with recycled aggregates, the greatest loss of elastic modulus was observed for RCFAC-100, a decrease of about 17%. This is consistent with the results of studies conducted by Pedro et al. [23] and Corinaldesi and Moriconi [64], which reported reductions in modulus of 20.4% and 21%, respectively.

The addition of 10 and 20% of RP caused only 2 and 6% of elastic modulus loss, showing superior modulus compared to other types of concrete, but a sharp loss of 23% was observed at 30% RP addition. Although this was not exactly consistent with the conclusion of a study by Xiao et al. [51], that replacing up to 30% of cement by RP had a minimal negative effect on the strength of concrete, the authors of the study noted that a significant reduction was observed at the RP replacement ratio of 45%.

## 4. Correlation between Properties of Concrete

### 4.1. Relationship between Compressive Strength and Density

**Figure 10:**
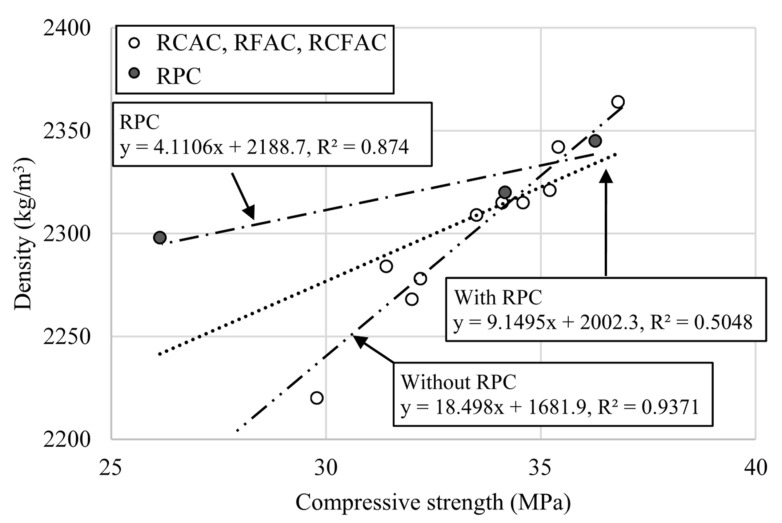
Correlation between compressive strength and density.

Figure 10 shows the correlation between compressive strength and density of concretes. A tendency to increase the compressive strength was observed as the density increased, but the coefficient of determination (R^2^) for all specimens was 0.50, which does not indicate a strong correlation. In particular, considering that RPC-30 mix has a very high density of about 2300 kg/m^3^ at the low strength of 26 MPa compared to other mixtures, regression analysis of concrete made with recycled aggregates (i.e., RCAC, RFAC, and RCFAC) and RP was performed separately. As a result, strong correlations were found in both groups. Concrete with RP showed a R^2^ of 0.87, and concrete with aggregates had a R^2^ of 0.94.

### 4.2. Relationship between Compressive Strength and Splitting Tensile Strength 

**Figure 11:**
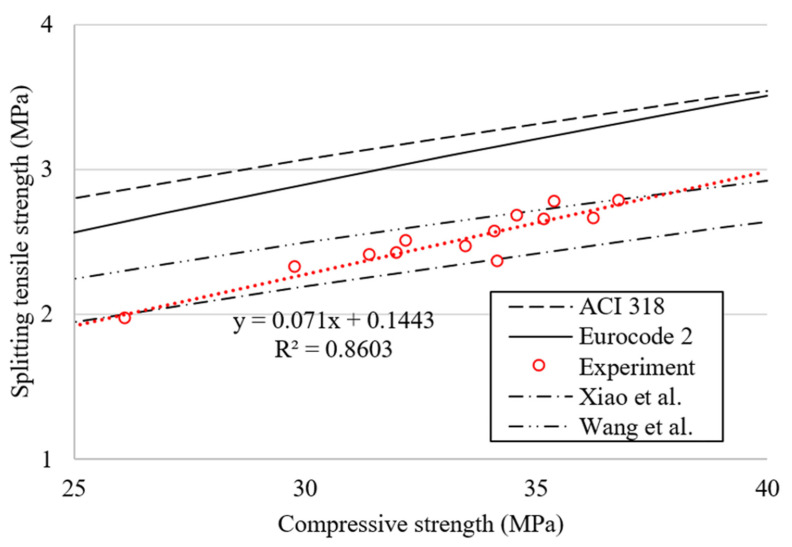
Correlation between compressive strength and tensile strength.

The relationship between compressive strength (*f_cu_*) and splitting tensile strength (*f_sp_*) at 28 days of age is shown in Figure 11. The compressive–tensile strength relationships presented in EN 1992-1-1 [65] and ACI 318-14 [66] (Equations (1) and (2)), and prediction models proposed by other researchers [67,68] (Equations (3) and (4)), were plotted for comparison with the current results.

(1) $$f_{sp}=0.3f_{cu}^{\left(\frac{2}{3}\right)}$$

(2) $$f_{sp}=0.56f_{cu}^{0.5}$$

(3) $$f_{sp}=0.24f_{cu}^{0.65}$$

(4) $$f_{sp}=1.49f_{cu}^{0.32}-1.93$$

Compressive strength and splitting tensile strength in this study showed a good correlation with a R^2^ of 0.86, but as can be seen in Figure 11, it is clear that the equations presented in the codes overestimated the splitting tensile strength. This may be because these two codes were not specifically developed with a focus on concrete made from recycled concrete materials. On the other hand, Equations (3) and (4) were derived based on concrete incorporating recycled aggregates, and are in good agreement with the experimental results of this study.

### 4.3. Relationship between Compressive Strength and Elastic Modulus 

**Figure 12:**
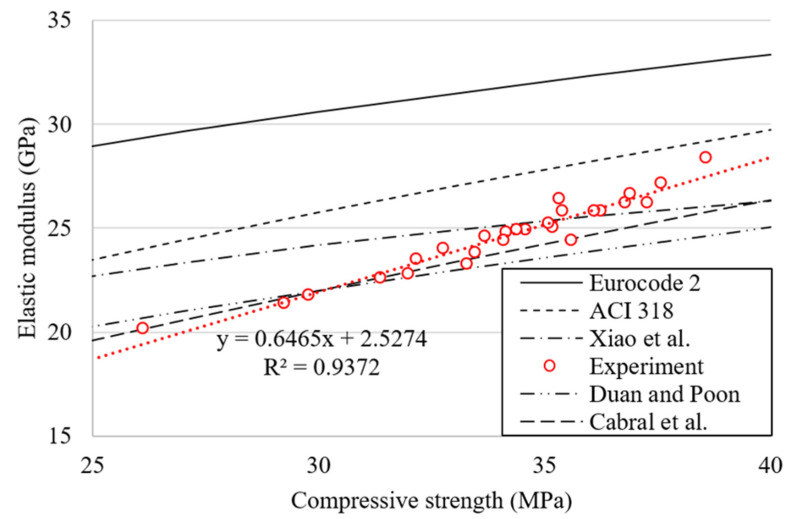
Correlation between compressive strength and elastic modulus.

Figure 12 shows the relationship between the compressive strength and the elastic modulus (*E_c_*) of concrete mixtures at 28 and 56 days. In Figure 12, the relationships presented in EN 1992-1-1 [65] and ACI 318-14 [66] codes (Equations (5) and (6)) and the compressive strength–modulus prediction model provided by other researchers [56,67,69] (Equations (7)–(9)) were also presented for comparison with the results of this study.

(5) $$\mathrm{Ec}=22{\left(f_{cu}/10\right)}^{0.3}$$

(6) $$\mathrm{Ec}=4700\sqrt{f_{cu}}$$

(7) $$\mathrm{Ec}=2.58f_{cu}^{0.63}$$

(8) $$\mathrm{Ec}=4.7863f_{cu}^{0.4485}$$

(9) Ec=102(2.8+40.1fcu)

A strong correlation with the R^2^ value of 0.94 was observed between compressive strength and elastic modulus. In addition, a similar pattern of the relationship between compressive strength and splitting tensile strength found in the previous section was observed.

The equations presented in each code overestimated the elastic modulus, while the prediction models based on concrete with recycled aggregates agreed well with the current experimental results. In this regard, Wang et al. [68] pointed out that the prediction models established based on NAC may no longer be suitable because the influence of recycled aggregates on the compressive strength and on the elastic modulus is different.

## 5. Cost Analysis and Environmental Impact Assessment

In Table 7, the manufacturing cost per cubic meter of all types of concrete and the 28-day compressive strength are provided along with the cost value and eco-efficiency. The cost value was obtained by dividing the 28-day compressive strength by the manufacturing cost, while the eco-efficiency was obtained by dividing the 28-day compressive strength by the GWP index. Both values are represented as relative value based on the RC value, i.e., RC has a value of 1.

All the concrete mixes incorporating recycled concrete materials are about 2.6% to 20% more economical than RC made of NCA and NFA, in proportion to the material replacement ratio. It should be noted that the concretes with the lowest manufacturing cost are RPC-30 and RCFAC-100, but these two mixtures did not achieve the target strength. Therefore, the unit price of concrete should not be considered as the only parameter in the economic analysis, and cost–benefit analysis should be performed based on the intended purpose. Since the slump of all concretes was within the tolerance range (100 ± 25 mm), a target strength of 30 MPa should be considered in this study. The same principle applies to eco-efficiency analysis. In view of that, the mixtures with values higher than RC (i.e., values greater than 1) were RPC-10 and RPC-20. Since the unit cost of OPC is more than ten times higher than that of aggregate, replacing OPC by RP even at a low replacement ratio has a higher value over replacing natural aggregates with recycled aggregates.

A similar pattern was observed in environmental impact assessments. It is clear that concrete made from recycled materials has a lower GWP than RC, providing environmental benefits. When RCA and RFA were used separately at 100%, GWP could be reduced by about 3–4%, and when both materials were used simultaneously, about 7% of GWP could be reduced. Moreover, concrete with 10% replacement of OPC with RP reduced GWP by 7%, which is similar to that of concrete incorporating 100% RCA and 100% RFA simultaneously. The environmental impact was reduced by up to 20% as the RP content was increased up to 30%. This is because the CO_2_ emissions from cement manufacturing were much higher than those from aggregate manufacturing. According to Flower and Sanjayan [4], the CO_2_ emission coefficient related to cement production is 0.82 t CO_2_-eq./ton, while those of coarse granite aggregates and fine aggregates are 0.0459 and 0.0139 t CO_2_-eq./ton, respectively. Therefore, the replacement of OPC by RP can provide greater environmental benefits than the replacement of natural aggregates by recycled aggregates. For this reason, in the eco-efficiency defined as the ratio of the 28-day compressive strength to the GWP, concretes that showed higher values than RC were RPC-10 and RPC-20, and the remaining mixtures were in the range of 0.87–0.97, showing lower values than RC.

Considering the above, the cost and environmental benefits of using recycled concrete materials are not sufficient to offset the unfavorable effect on the mechanical performance of concrete in some cases. Nevertheless, it should be noted that all concretes, except for RPC-30 and RCFAC-100, achieved their target strength and thus could be applied for structural purposes. In terms of cost value and eco-efficiency, the addition of 20% RP is recommended. In addition, although the cost value and eco-efficiency were lower than those of RC, the separate uses of 100% RCA and 100% RFA can be considered as a viable option because the unit cost and GWP of RCAC-100 and RFAC-100 were lower than those of RC while satisfying the required compressive strength. When RCA and RFA are used simultaneously, the replacement rate can be up to 60%.

## 6. Conclusions

A study was conducted on the properties and the economic and environmental impact of concrete incorporating recycled concrete materials of different size fractions obtained from concrete waste. The following conclusions can be drawn:

- With the increased replacement ratios of natural materials by recycled materials, the slump of the concrete mixes was reduced (up to 19%) and the air content was increased (up to 0.6%) compared to the reference concrete, but the fresh properties were within the range required by the standard.
- As the replacement ratio increased, the mechanical properties of concrete decreased. The properties decreased in the order of RFAC, RCAC, and RCFAC at the same replacement ratio.
- The reduction of compressive strength and elastic modulus was only 1–4% for concrete with 10% RP and 6–8% for concrete with 20% RP. However, when 30% RP was added, the mechanical properties showed a rapid decrease of 23~29%, thus special attention is required for its use. Nevertheless, all mixtures could be applied as structural concretes under different environmental exposure conditions.
- The relationship between compressive strength, elastic modulus, and splitting tensile strength of concrete containing different size fractions of recycled concrete materials showed a strong correlation. However, for the relationship between compressive strength and density, RPC needs to be considered separately from RCAC, RFAC, and RCFAC.
- Replacing OPC with RP by up to 20%, cost value and eco-efficiency were superior to those of RC. Although the cost value and eco-efficiency of concrete incorporating RCA and RFA were lower, the production cost and GWP were lower than those of RC; thus, it can be considered economical and eco-friendly if the intended requirements are achieved.

## Figures and Tables

**Table 1:** Mix proportion of reference concrete.

Mix	OPC (kg/m^3^)	Water (kg/m^3^)	w/c	NCA (kg/m^3^)	NFA (kg/m^3^)
RC	389	175	0.45	1011	740

**Table 2:** Physical properties of aggregates.

Aggregates	Specific Gravity	Water Absorption (%)
NCA	2.68	0.88
RCA	2.41	4.45
NFA	2.60	0.91
RFA	2.22	5.38

**Table 3:** Mix proportions for concrete with recycled aggregates and recycled powder.

Mix Designation	OPC (kg/m^3^)	RP (kg/m^3^)	Water (kg/m^3^)	w/c	NCA (kg/m^3^)	RCA (kg/m^3^)	NFA (kg/m^3^)	RFA (kg/m^3^)
RCAC-30	389	0	175	0.45	707	279	740	0
RCAC-60	389	0	175	0.45	404	545	740	0
RCAC-100	389	0	175	0.45	0	909	740	0
RFAC-30	389	0	175	0.45	1011	0	518	189
RFAC-60	389	0	175	0.45	1011	0	296	379
RFAC-100	389	0	175	0.45	1011	0	0	632
RPC-10 [32]	350	39	175	0.45	1011	0	740	0
RPC-20 [32]	311	78	175	0.45	1011	0	740	0
RPC-30 [32]	272	117	175	0.45	1011	0	740	0
RCFAC-30	389	0	175	0.45	707	279	518	189
RCFAC-60	389	0	175	0.45	404	545	296	379
RCFAC100 [32]	389	0	175	0.45	0	909	0	632

**Table 6:** Environmental exposure conditions based on compressive strength of concrete.

Concrete Grade	Exposure Class *	Applicable Mix
C8/10	X0—no risk of corrosion	-
C16/20	XC1—dry or permanent wet XC2—wet, rarely dry	-
C20/25	XC3—moderate humidity XC4—cyclic wet and dry	-
C25/30	XF2—moderate water absorption (saturation), water includes de-icing agent	RPC-30 RCFAC-100
C30/37	XD1—moderately wet XD2—wet, occasionally dry XS1—action of salts in air = atmosphere XF1—moderate water absorption (saturation) XF3—strong water absorption (saturation), water without de-icing agent XF4—strong water absorption (saturation), water includes de-icing agent XA1—weak chemical aggression XA2—moderate chemical aggression XM1—moderate risk of abrasion XM2—strong risk of abrasion	RCAC-60 RCAC-100 RFAC-60 RFAC-100 RPC-20 RCFAC-30 RCFAC-60
C35/45	XD3—moderately wet and dry XS2—permanent immersion in water XS3—tidal, splash and aerosol zones XA3—strong chemical aggression XM3—extreme risk of abrasion	RC RCAC-30 RFAC-30 RPC-10

* X0—no risk of corrosion; XC—corrosion caused by carbonation; XD—corrosion caused by chloride except sea water chloride; XS—corrosion caused by sea water chloride; XF—freezing–thawing attack; XA—chemical attack; XM—abrasion.

**Table 7:** Cost and environmental analysis of various concretes.

Mix	Cost (USD/m^3^)	GWP (kg CO_2_-eq./m^3^)	Compressive Strength (MPa)	Cost Value	Eco-Efficiency	Target Strength
RC	56.20	405.22	36.8	1	1	Pass
RCAC-30	54.73	399.88	35.2	0.98	0.97	Pass
RCAC-60	53.20	394.47	33.5	0.96	0.93	Pass
RCAC-100	51.21	387.32	31.4	0.94	0.89	Pass
RFAC-30	55.33	401.94	35.4	0.98	0.97	Pass
RFAC-60	54.47	398.67	34.6	0.97	0.96	Pass
RFAC-100	53.31	394.31	32.2	0.92	0.90	Pass
RPC-10	52.33	378.60	36.3	1.06	1.06	Pass
RPC-20	48.45	351.87	34.2	1.08	1.07	Pass
RPC-30	44.56	325.15	26.1	0.90	0.89	Fail
RCFAC-30	53.85	396.60	34.1	0.97	0.95	Pass
RCFAC-60	51.46	387.92	32.0	0.95	0.91	Pass
RCFAC-100	48.32	376.40	29.8	0.94	0.87	Fail

## Data Availability

Not applicable.
